# Editorial: Deciphering host-gut microbiota communication in immunity and disease

**DOI:** 10.3389/fnut.2023.1178039

**Published:** 2023-03-27

**Authors:** Jian Tan, Severine Navarro, Laurence Macia

**Affiliations:** ^1^Charles Perkins Centre, The University of Sydney, Sydney, NSW, Australia; ^2^Faculty of Medicine and Health, School of Medical Sciences, The University of Sydney, Sydney, NSW, Australia; ^3^QIMR Berghofer Institute of Medical Research, Herston, QLD, Australia; ^4^Faculty of Health, Centre for Childhood Nutrition Research, Queensland University of Technology, Brisbane, QLD, Australia; ^5^Faculty of Medicine, The University of Queensland, Herston, QLD, Australia; ^6^Sydney Cytometry, The University of Sydney and The Centenary Institute, Sydney, NSW, Australia

**Keywords:** gut microbiota, prebiotic, probiotic, postbiotic, microbiome and dysbiosis, dietary fiber

The rise in non-communicable diseases over the last two decades highlights the environment as a major risk factor. Changes in dietary habits in western countries have significantly reshaped the composition of the gut microbiota. This has resulted in the loss of gut bacterial species that have co-evolved with humans for millennia and changing the balance of microbial-derived metabolites crucial for maintaining the symbiotic relationship between gut bacteria and their host.

Non-communicable diseases are commonly associated with a detrimental shift in the gut microbiota composition known as dysbiosis. Dysbiosis contributes to increased gut permeability and overexposure of the host to microbial products, which triggers inflammation. Targeting the gut microbiota to restore a healthy gut environment and to control the immune response is thus an attractive strategy to treat inflammatory diseases. There are different strategies to target the gut microbiota, either by introducing a healthy gut microbiota *via* fecal microbiota transplantation, by reshaping the gut microbiota through dietary intervention (prebiotics), by introducing beneficial bacteria (probiotics) or by administering by-products released by healthy gut microbiota (postbiotics).

Dietary fiber, a prebiotic, is a complex carbohydrate indigestible to the host but represents a major source of energy for gut bacteria. Corrêa et al. highlighted that the beneficial reshaping of the gut microbiota by dietary fiber reduced the development of pulmonary diseases, including COPD, pulmonary fibrosis, allergic asthma, lung cancer, and acute respiratory distress syndrome in mice, confirming that targeting the gut microbiota could improve host's health.

Zeng et al. presented the effects of probiotics in six different types of arthritis through a meta-analysis of 34 randomized clinical trials. Inflammatory arthritis is characterized by chronic joint inflammation as well as dysbiosis and increased gut permeability. A wide range of probiotics was tested mostly from the *Lactobacillus* and *Bifidobacterium* genera. The main conclusion of this analysis is that probiotics are generally safe for use in humans but the lack of reproducibility and the variability between trials make it inconclusive whether probiotics are effective for arthritis. Some potential benefits of probiotics were reported in gout, osteoporosis and osteoarthritis, while no benefits were observed in spondyloarthritis and juvenile idiopathic arthritis. The inconsistency of response included either decreased inflammatory markers (CRP), decreased swollen joints or no differences with mild adverse events reported by a few patients. This variability may be explained by the intrinsic differences in the gut microbiota of the patients. A study has shown that people could be subclassified as responders or non-responders depending on the engraftment ability of the probiotics (1), but there is currently no method to predict an individual's response. A personalized approach to probiotic intervention would likely give more consistent results and help determine whether probiotics could be efficient therapeutics in inflammatory diseases, such as arthritis. Alternatively, Fernandes Rodrigues et al. reported the role of a novel class of probiotics. These probiotics have mucophagic properties, extracting their energy by consuming the host's mucus. They present an overview of the clinical benefits of *Akkermancia municiphila* in inflammatory bowel disease, type 2 diabetes and metabolic syndrome. These gram-negative bacteria produce the short-chain fatty acids acetate and propionate, which modulate host immune function and support a healthy gut microbiota through the colonization of beneficial bacteria.

Postbiotic intervention is another strategy to improve the host's health, acting independently of the gut microbiota and reducing interindividual response variability. While postbiotics show promise in preclinical studies, their efficacy in the clinic is poorly known. Preclinical studies show the benefit of short-chain fatty acids, by-products of dietary fiber fermentation, in pulmonary diseases like COPD, pulmonary fibrosis, allergic asthma, lung cancer, and acute respiratory distress syndrome in mice. SCFA also promote anti-inflammatory regulatory T cell generation, gut homeostasis and increase bone density. Rheumatoid arthritis (RA) patients had lower or unchanged levels of SCFA and preclinical studies have shown potential benefits of dietary fiber and SCFA in RA. Xu et al. also presented the therapeutic potential of gut microbiota-modified secondary bile acids (BA), particularly deoxycholic acid and lithocholic acid and derivatives in suppressing IL-6 production by macrophages, which is elevated in RA.

Extensive work in preclinical models showed that conjugated BA promoted anti-inflammatory regulatory T cells while inhibiting Th17 cells. Of note, not all conjugated BA have anti-inflammatory effects, with some having pro-Th17 effects discussed elsewhere (2). While some evidence shows the impact of conjugated BA on human Treg generation *in vitro*, their role in RA patients may be limited as a study has shown that TGR5, a bile acid receptor, is downregulated in PBMC of patients with RA (3). L-tryptophan is an essential amino acid that can be metabolized by the host or gut bacteria through the indole pathway, the kynurenine pathway or the serotonin pathway that supports gut integrity. L-tryptophan derivatives generated by the indole pathway can support gut homeostasis and support Treg development, with lower indole derivatives observed in the synovial fluid of RA patients. Kynurenine on the other hand prevents bone loss. This suggests that different L-tryptophan derivatives can improve different aspects of RA, which needs to be proven in humans. Riazati et al. examined the association between L-tryptophan-derived metabolite levels and immune profile in an observational study involving 362 volunteers. Overall, the microbial-derived L-tryptophan metabolites, indole, indole acetic acid and indole propionic acid were not associated with any immune markers. In contrast, the host-derived L-tryptophan metabolite, kynurenine, was associated with plasma inflammatory markers, particularly IFN-γ-induced protein-10, neopterin (an IFN-γ-induced proinflammatory factor) and TNF. They also mention a lack of association between protein intake and microbial L-tryptophan-derived metabolites that could be due to host metabolism, sex or gut microbiota composition.

Other than metabolites, proteins, outer-membrane vesicles or even pasteurized organisms may be more beneficial than live probiotics. Fernandes Rodrigues et al. showed that *A. municiphila*-derived proteins were more beneficial than the live bacteria, opening safer and more controlled therapeutic opportunities.

Altogether, these articles highlight the gut microbiota as a promising therapeutic target for numerous diseases. Their efficacy in humans and the parameters that may influence their efficacy, including microbiota composition, genetics, gender and type of disease, are largely undefined. A holistic understanding is needed to optimize their therapeutic potential.

## Author contributions

All authors listed have made a substantial, direct, and intellectual contribution to the work and approved it for publication.
